# Post-gel polymerisation shrinkage profiling of polymer biomaterials using a chirped fibre Bragg grating

**DOI:** 10.1038/s41598-020-80838-5

**Published:** 2021-01-14

**Authors:** Ginu Rajan, Alex Wong, Paul Farrar, Gangadhara B. Prusty

**Affiliations:** 1 School of Electrical, Computer and Telecommunications Engineering, University of Wollongong, Wollongong, NSW 2522 Australia; 2 SDI Ltd, Bayswater, VIC 3153 Australia; 3 ARC Training Centre for Automated Manufacture of Advanced Composites, UNSW Sydney, Sydney, NSW 2052 Australia

**Keywords:** Biomaterials, Optical sensors

## Abstract

A strain profile measurement technique using a chirped fibre Bragg grating (CFBG) sensor by implementing an integration of differences (IOD) method is reported in this paper. Using the IOD method the spatial distribution of strain along the length of the CFBG is extracted from its power reflectance spectra. As a proof of concept demonstration, the developed technique is applied to measure the polymerisation shrinkage strain profile of a photo-cured polymer dental composite which exhibits a non-uniform strain distribution attributed to the curing lamp characteristics. The result from the CFBG technique is compared with that of an FBG array embedded in the dental composite and is correlated with the degree of conversion of the material which also depends on the curing lamp intensity distribution. This technology will have significant impact and applications in a range of medical, materials and engineering areas where strain or temperature gradient profile measurement is required in smaller scales.

## Introduction

Polymer biomaterials usage continues to grow in medical and dentistry applications^1^. They offer advantages in manipulability and application adaptability, without the drawbacks associated with metallic materials. One of the current widely accepted initiation modes for hardening process required for such polymer dental composites is the photo-polymerisation method^2^. In order to facilitate the growing demands for longevity and durability of polymer dental materials, studying the mechanical stresses that develop within the material during curing and lifespan proves significant, of which the polymerisation shrinkage (PS) is important. Molecular densification and related macroscopic effects of PS strain and/or stress influences the material’s post-cure characteristics. Knowing the polymerisation evolution across the material in real time can lead to a better understanding of curing kinetics which can be translated into optimised material formulations with reduced PS and post curing effects/defects^3–8^.

PS is a vector quantity and PS strain/stress distribution across a material depends on the excitation/initiation mode and the shrinkage pattern is often anisotropic^9^. Different approaches were developed to manage the shrinkage of biomaterials, such as low modulus intermediate materials, different polymerisation initiation mechanisms and alternative excitation methods to delay curing kinetics^10–14^. Due to the complex mechanisms involved in the material formulation, a material ‘shrinkage profile’ as opposed to an average measurement, can be used to establish a correlation between curing mechanisms, excitation sources and additive dispersion, leading towards better material development. Traditional PS measurement systems merely provide an overall PS measurement for tested samples, but the true PS may vary within the sample^15^. It has long been known that the PS and degree of conversion (DC) are correlated for photo-cured dental composites and is also non-uniform but only recent developments in measurement techniques have directly revealed such non-uniformity^16,17^.

One of the existing methods for PS profile measurement is the digital image correlation technique where the before-and-after PS of the material is measured, but faces limitations in time and strain resolution due to the nature of the technique relying on visually tracking physical movements on the sample surface^18^. Thus, the true and real-time shrinkage mapping of polymer biomaterials and the subsequent understanding of the PS evolution across the material and its dependence on the curing excitation method or material composition is still an unsolved problem. Optical fibre sensing (OFS) technology which provides novel solutions to many challenging instrumentation requirements in a variety of applications could be a potential solution to this problem^19^. Among the various types of OFSs available, fibre Bragg grating (FBG) is the preferred sensor for composite materials due to ease of embedding the sensor within the material^20,21^. Traditional PS measurements provide the total PS of the material, whereas FBG based technique only provides the post-gel PS which is more clinically significant. FBG based approach is demonstrated for real-time post-gel PS measurement of dental materials with good accuracy but provides only singular measurements per sensor^22,23^. The results show that FBG measures post-gel PS strain comparable with strain gauges for the tested commercial dental composites. To transition further into the spatial PS regime, FBG arrays are used^17^, but it is not viable to scale up the number of FBGs since the excess optical fibres in the materials would affect the characteristics properties of the material itself.

In this study, we explore the use of a linearly chirped fibre Bragg grating (CFBG) as a post-gel PS profiling sensor with a significant increase in spatial resolution, with excellent time and strain resolution. In a linear CFBG, the grating period and the resultant Bragg wavelength varies linearly along the grating axis, and as a result its reflection spectrum is broader, typically in the range of tens of nanometres^24^. CFBGs are commonly used in optical communications^24–26^, but its use in sensing applications is also enormous, ranging from structural health monitoring to biomedical engineering^27–29^. Various interrogation systems have been also developed for CFBGs to measure physical parameters such as strain and temperature^30^. In this paper a novel integration of differences (IOD) method and data processing algorithm are developed and applied to a CFBG sensor, vastly improving the accuracy in measuring the spatial distribution of strain along its length compared to FBG sensor arrays. This technique is applied to measure the PS profile of a polymer dental composite in real time where the outcome facilitates for better formulation, characterisation and curing of polymer composites/materials. This unique curing evaluation method will have an impact on a wide range of applications/end users from medicine to dentistry to engineering.

## Methods

### CFBG strain profiling using an integration of differences method

Integration of differences methods for CFBG interrogation rely on the reflection intensity-spectrum of the CFBG^31^. Whilst other methods of CFBG interrogation have been developed by other groups^30,32,33^, IOD methods benefit from simplicity by removing the need for neither a sophisticated setup nor detailed knowledge of the CFBG in use. The proposed IOD method compares the reflection spectrum of a strain/temperature perturbed CFBG against its reference reflection spectrum measured prior to being perturbed. The difference is used to determine the variation of the one-dimensional perturbation along the length of the CFBG and edge-point values are determined using the wavelength shift of the 3 dB edges in the CFBG’s reflection spectrum, which are then used to fit the strain/temperature variation to the values at the boundaries.

The reflected Bragg wavelength of an ideal linearly chirped FBG along the x-axis (light propagation direction) can be described as^34^:

1 $${\uplambda }_{\upbeta }\left(x\right)=2{n}_{eff}\left({\Lambda }_{0}+Cx\right)+{\mathrm{\alpha }}_{\upepsilon ,\Delta T}\left(x\right)\left[\begin{array}{c}\upepsilon \left(x\right)\\\Delta T\left(x\right)\end{array}\right]=2{n}_{eff}{\Lambda }_{0}+\upbeta x+\mathrm{\alpha }\left(x\right)S\left(x\right),$$

where $\upbeta =2{n}_{eff}C$ with $C$ the coefficient of the linear chirp, ${n}_{eff}$ is the effective refractive index, ${\mathrm{\alpha }}_{\upepsilon ,\Delta T}$ the strain/temperature sensitivity, and $S\left(x\right)$ the subjected strain/temperature along the grating. Reiterating (1) as position in terms of Bragg wavelength:

2 $$x\left({\uplambda }_{\upbeta }\right)=\frac{1}{\upbeta }\left[ {\uplambda }_{\upbeta }-2{n}_{eff}{\Lambda }_{0}-\mathrm{\alpha }\left(x\left({\uplambda }_{\upbeta }\right)\right)S\left(x\left({\uplambda }_{\upbeta }\right)\right) \right],$$

The change in dimension size of *x* due to strain/perturbation is assumed negligible and thus *x* is treated as a common variable between the unperturbed and perturbed grating. Defining notations for the unperturbed (*U*) and strained/perturbed (*S*) cases for a given grating, if we consider ${\uplambda }_{U}$ and ${\uplambda }_{S}$ for the *U* and *S* cases respectively, and denoting ${x}_{U}$ and ${x}_{S}$ for their respective definitions then leading off (1) and (2) gives:

3a $${x}_{U}\left({\uplambda }_{U}\right)=\frac{1}{\upbeta }\left[ {\uplambda }_{U}-2{n}_{eff}{\Lambda }_{0} \right],$$

3b $${x}_{S}\left({\uplambda }_{S}\right)=\frac{1}{\upbeta }\left[ {\uplambda }_{S}-2{n}_{eff}{\Lambda }_{0}-\mathrm{\alpha }\left({x}_{S}\left({\uplambda }_{S}\right)\right)S\left({x}_{S}\left({\uplambda }_{S}\right)\right) \right],$$

3c $${\uplambda }_{U}\left({x}_{U}\right)=2{n}_{eff}{\Lambda }_{0}+\upbeta {x}_{U},$$

3d $${\uplambda }_{S}\left({x}_{S}\right)=2{n}_{eff}{\Lambda }_{0}+\upbeta {x}_{S}+\mathrm{\alpha }\left({x}_{S}\right)S\left({x}_{S}\right),$$

Assuming a constant strain/temperature sensitivity $\mathrm{\alpha }$, and defining $S\left({x}_{S}\right)\equiv S\left({x}_{S}\left({\uplambda }_{S}\right)\right)$ it follows that:

4a $$\frac{\Delta {x}_{U}\left({\uplambda }_{U}\right)}{\Delta {\uplambda }_{U}}\approx \frac{\mathrm{d}{x}_{U}\left({\uplambda }_{U}\right)}{d{\uplambda }_{U}}=\frac{1}{\upbeta },$$

4b $$\frac{\Delta {x}_{S}\left({\uplambda }_{S}\right)}{\Delta {\uplambda }_{S}}\approx \frac{\mathrm{d}{x}_{S}\left({\uplambda }_{S}\right)}{d{\uplambda }_{S}}=\frac{1}{\upbeta }\left[1-\mathrm{\alpha }\frac{\mathrm{d}S\left({x}_{S}\left({\uplambda }_{S}\right)\right)}{\mathrm{d}{\uplambda }_{S}}\right]=\frac{1}{\upbeta }\left[1-\mathrm{\alpha }{\upomega }_{\uplambda }\right],$$

4c $$\frac{\Delta {\uplambda }_{U}\left({x}_{U}\right)}{\Delta {x}_{U}}\approx \frac{\mathrm{d}{\uplambda }_{U}\left({x}_{U}\right)}{\mathrm{d}{x}_{U}}=\upbeta ,$$

4d $$\frac{\Delta {\uplambda }_{S}\left(x\right)}{\Delta {x}_{S}}\approx \frac{\mathrm{d}{\uplambda }_{S}\left({x}_{S}\right)}{\mathrm{d}{x}_{S}}=\upbeta +\mathrm{\alpha }\frac{\mathrm{d}S\left({x}_{S}\right)}{\mathrm{d}{x}_{S}}=\upbeta +\mathrm{\alpha }{\upomega }_{x},$$

where we define the strain/temperature gradients: ${\upomega }_{x}=\frac{\mathrm{d}S\left({x}_{S}\right)}{\mathrm{d}{x}_{S}}$ and ${\upomega }_{\uplambda }=\frac{\mathrm{d}S\left({x}_{S}\left({\uplambda }_{S}\right)\right)}{\mathrm{d}{\uplambda }_{S}}$.

The reflectivity $R\left({\uplambda }_{\upbeta }\right)$ as a function of wavelength is obtained using the measured reflection spectrum from an optical spectrum analyser. It is then transformed to ‘coupling strength–grating length’ product units by the following:

5 $$Y\left({\uplambda }_{\upbeta }\right)=tan{h}^{-1}\left(\sqrt{R\left({\uplambda }_{\upbeta }\right)}\right)\propto \frac{\Delta x\left({\uplambda }_{\upbeta }\right)}{\Delta {\uplambda }_{\upbeta }}\approx A\left(x\left({\uplambda }_{\upbeta }\right)\right)\frac{dx\left({\uplambda }_{\upbeta }\right)}{d{\uplambda }_{\upbeta }},$$

where $A\left(x\left({\uplambda }_{\upbeta }\right)\right)$ is an arbitrary variation factor that accounts for amplitude and grating-strength variation along the grating.

Variation in the intensity-spectrum independent to the wavelength-shift of the CFBG from sources such as amplitude variation from the light source, connection losses and fibre bend losses are assumed to be static and have not been accounted for in the formulation.

The following requires that ${x}_{U}\left({\uplambda }_{U}\right)={x}_{S}\left({\uplambda }_{S}\right)=x$ such that the mapping {${\uplambda }_{U}\leftrightarrow x\leftrightarrow {\uplambda }_{S}\}$ exists, which thus requires:

6a $${\uplambda }_{S}\left(x\right)={\uplambda }_{U}\left(x\right)+\mathrm{\alpha }S\left(x\right),$$

6b $$\frac{d{\uplambda }_{U}}{d{\uplambda }_{S}}=1-\mathrm{\alpha }{\upomega }_{\uplambda } ,$$

Defining *U* and *S* measurements respectively, we have ${Y}_{U|S}^{\uplambda }\equiv {Y}_{U|S}^{\uplambda }\left({\uplambda }_{U|S}\right)\equiv {Y}_{U|S}^{\uplambda }\left({\uplambda }_{U|S}\left(x\right)\right)$ as functions of their Bragg wavelengths expressed in *x*, and ${Y}_{U|S}^{x}\equiv {Y}_{U|S}^{x}\left(x\right)\equiv {Y}_{U|S}^{x}\left(x\left({\uplambda }_{U|S}\right)\right)$ as functions of *x* expressed in their Bragg wavelengths. From applying (5) to (4a–d), it follows that:

7a $$\frac{{Y}_{S}^{x}-{Y}_{U}^{x}}{{Y}_{U}^{x}}=-\mathrm{\alpha }{\upomega }_{\uplambda },$$

7b $$\frac{{Y}_{S}^{x}-{Y}_{U}^{x}}{{Y}_{S}^{x}}=\frac{-\mathrm{\alpha }{\upomega }_{\uplambda }}{1-\mathrm{\alpha }{\upomega }_{\uplambda }},$$

7c $$\frac{{Y}_{S}^{\uplambda }-{Y}_{U}^{\uplambda }}{{Y}_{U}^{\uplambda }}= \frac{-\mathrm{\alpha }{\upomega }_{x}}{\upbeta +\mathrm{\alpha }{\upomega }_{x}},$$

7d $$\frac{{Y}_{S}^{\uplambda }-{Y}_{U}^{\uplambda }}{{Y}_{S}^{\uplambda }}= -\frac{\mathrm{\alpha }}{\upbeta }{\upomega }_{x},$$

Integrating (7a–d) against wavelength using (4a–d) and (6b) as substitutions, the strain/temperature perturbation can be recovered as follows:

8a $$-\frac{1}{\mathrm{\alpha }}\underset{{\uplambda }_{0}}{\overset{\uplambda }{\int }}\frac{{Y}_{S}^{x}-{Y}_{U}^{x}}{{Y}_{U}^{x}}\mathrm{d}{\uplambda }_{S}=\underset{{\uplambda }_{0}}{\overset{\uplambda }{\int }}{\upomega }_{\uplambda }\mathrm{d}{\uplambda }_{S}=S\left(x\right),$$

8b $$-\frac{1}{\mathrm{\alpha }}\underset{{\uplambda }_{0}}{\overset{\uplambda }{\int }}\frac{{Y}_{S}^{x}-{Y}_{U}^{x}}{{Y}_{S}^{x}}\mathrm{d}{\uplambda }_{U}=\underset{{\uplambda }_{0}}{\overset{\uplambda }{\int }}\frac{{\upomega }_{\uplambda }}{1-\mathrm{\alpha }{\upomega }_{\uplambda }}\left(1-\mathrm{\alpha }{\upomega }_{\uplambda }\right)\mathrm{d}{\uplambda }_{S}=\underset{{\uplambda }_{0}}{\overset{\uplambda }{\int }}{\upomega }_{\uplambda }\mathrm{d}{\uplambda }_{S}=S\left(x\right),$$

8c $$-\frac{1}{\mathrm{\alpha }}\underset{{\uplambda }_{0}}{\overset{\uplambda }{\int }}\frac{{Y}_{S}^{\uplambda }-{Y}_{U}^{\uplambda }}{{Y}_{U}^{\uplambda }}\mathrm{d}{\uplambda }_{S}=\underset{{\uplambda }_{0}}{\overset{\uplambda }{\int }}\frac{{\upomega }_{x}}{\upbeta +\mathrm{\alpha }{\upomega }_{x}}\mathrm{d}{\uplambda }_{S}=\underset{{x}_{0}}{\overset{x}{\int }}\frac{{\upomega }_{x}}{\upbeta +\mathrm{\alpha }{\upomega }_{x}}\left(\upbeta +\mathrm{\alpha }{\upomega }_{x}\right)\mathrm{d}x=\underset{{x}_{0}}{\overset{x}{\int }}{\upomega }_{x}\mathrm{d}x=S\left(x\right)$$

8d $$-\frac{1}{\mathrm{\alpha }}\underset{{\uplambda }_{0}}{\overset{\uplambda }{\int }}\frac{{Y}_{S}^{\uplambda }-{Y}_{U}^{\uplambda }}{{Y}_{S}^{\uplambda }}\mathrm{d}{\uplambda }_{U}=\frac{1}{\upbeta }\underset{{\uplambda }_{0}}{\overset{\uplambda }{\int }}{\upomega }_{x}\mathrm{d}{\uplambda }_{U}=\frac{1}{\upbeta }\underset{{x}_{0}}{\overset{x}{\int }}{\upomega }_{x}\mathrm{\beta d}x=\underset{{x}_{0}}{\overset{x}{\int }}{\upomega }_{x}\mathrm{d}x=S\left(x\right),$$

Since the typical experimental data ${Y}_{U}^{x}\left(x\left({\uplambda }_{U}\right)\right)$ and ${Y}_{S}^{x}\left(x\left({\uplambda }_{S}\right)\right)$ exists in wavelength domain, (8a) and (8b) are used to perform the integration numerically, where (8a) is integrated against ${\uplambda }_{S}$ and (8b) against ${\uplambda }_{U}$. By treating the edge-points like regular FBGs, the strain/temperature at the endpoints is used to re-fit the integral result to match the endpoint on both sides, using a linear offset. The $\{{\uplambda }_{S}\leftrightarrow x\}$ map is required to form the ${\{\uplambda }_{U}\leftrightarrow x\leftrightarrow {\uplambda }_{S}\}$ map for the integration but since the perturbation remains unknown, the $\{{\uplambda }_{S}\leftrightarrow x\}$ map is also unknown and is given an initial 1-to-1 map between the two. By setting the number of data points or segments in ${\uplambda }_{U}$ and ${\uplambda }_{S}$ as the same, each step along the integration corresponds to the same step in $x$ for both ${Y}_{U}^{x}$ and ${Y}_{S}^{x}$ if $\{{\uplambda }_{S}\leftrightarrow x\}$ is known. The result from the integration of (8a) and (8b) will have slightly different results due unknown $\{{\uplambda }_{S}\leftrightarrow x\}$. The results of both are averaged to create the result of the first integration which we shall denote ${S}_{1}\left(x\right)$, which is used to remap $\{{\uplambda }_{S}\leftrightarrow x\}$ via (6a) such that:

9 $${\uplambda }_{S\, new}\left(x\right)={\uplambda }_{U}\left(x\right)+\mathrm{\alpha }{S}_{1}\left(x\right),$$

From (9) the new map is given as follows:

$$\mathrm{new\, map} :\{x\leftrightarrow {\uplambda }_{S\mathrm{ new}}\}$$

$$\mathrm{define}\,{x}_{\mathrm{new}} :\{{x}_{\mathrm{new}}\leftrightarrow {\uplambda }_{S}\} = \{x\leftrightarrow {\uplambda }_{S\mathrm{ new}}\}$$

The new position variable ${x}_{\mathrm{new}}$ is then used to remap the ${Y}_{S}$ values. The values are forced back into the same original position variable as opposed to using the new position:

$$\mathrm{new\, map} :\{{Y}_{S}\leftrightarrow {x}_{\mathrm{new}}\}$$

$$\mathrm{define} {Y}_{S\mathrm{ new}} : \{{Y}_{S\mathrm{ new}}\leftrightarrow x\}={\{Y}_{S}\leftrightarrow {x}_{\mathrm{new}}\}$$

**Figure 1:**
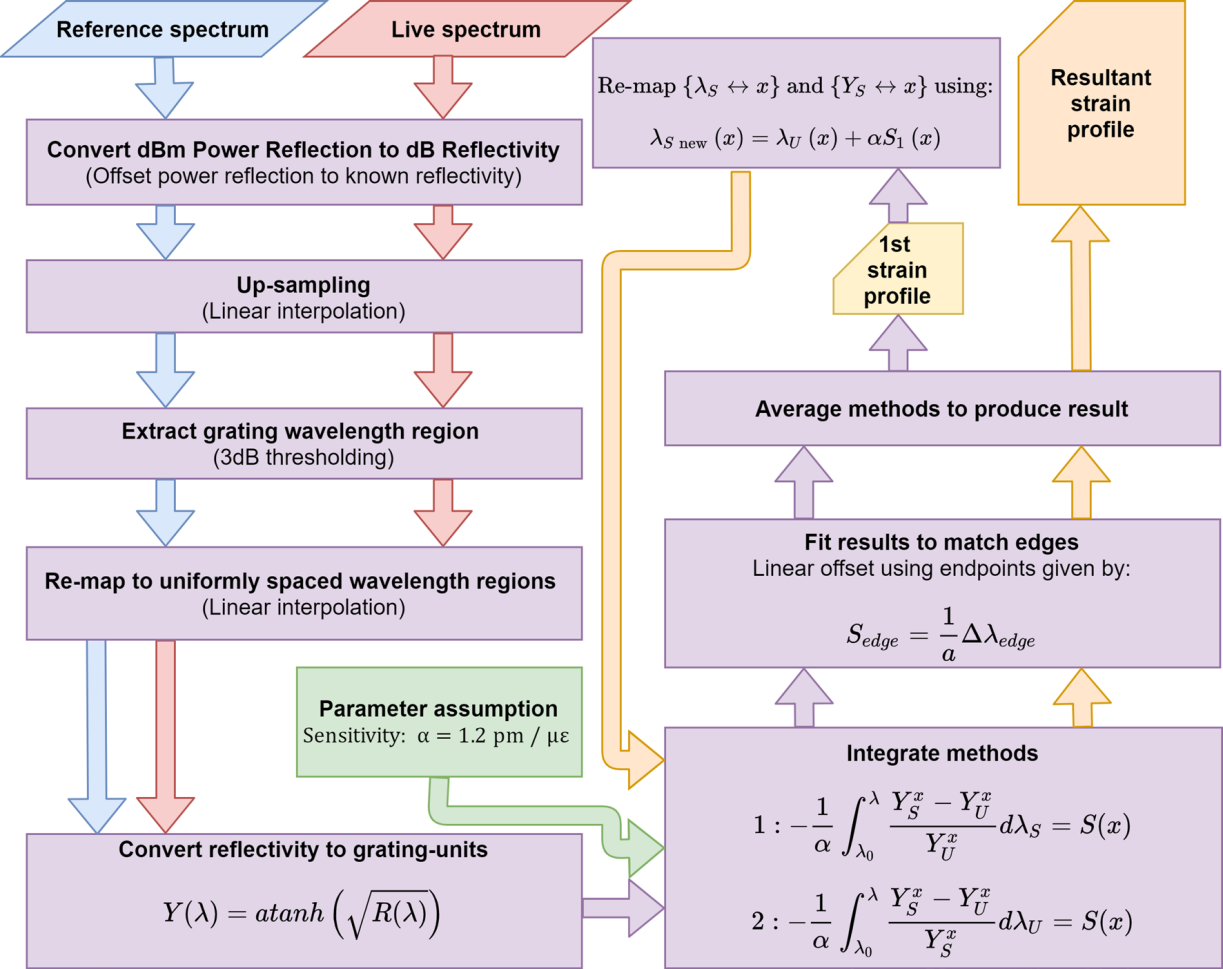
A flowchart of the process involved in measuring strain/temperature profile from a CFBG.

This ensures the position variable remains shared between the unperturbed and perturbed data during the integration. Subsequently, ${Y}_{S\mathrm{ new}}$ and ${\uplambda }_{S new}$ replace their old counterparts in integrating (8a) and (8b). The outcome of both are averaged to produce a more accurate result. This process can be repeated indefinitely, but in practice, appear to converge extremely slowly so only one additional pass is necessary. The whole IOD process and data processing is demonstrated in Fig. 1.

### Experimental arrangement to measure the strain profile of a dental composite using the developed method

The capability of the CFBG to measure the strain profile accurately by implementing the IOD approach is demonstrated through a dental composite PS profile measurement. The test material used in the study was a commercial photo-cured resin-based composite Beautifil FO3 (from Shofu Inc.). As the polymer degree of conversion and the resultant PS of the material depend on the intensity profile of the curing lamp, we aim to measure resultant PS which could reflect the characteristics of the intensity distribution of the curing lamp.

**Figure 2:**
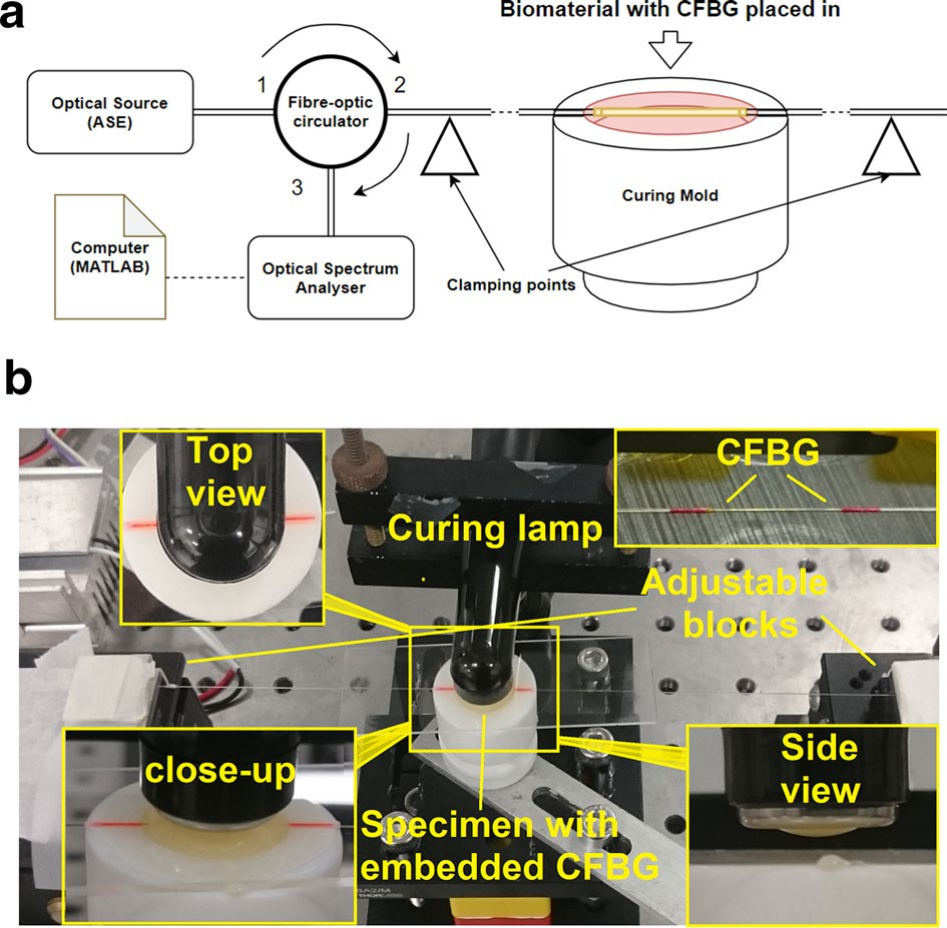
(**a**) Schematic experimental arrangement, (**b**) photograph of the setup.

The CFBGs used are 10 mm long, centred at 1550 nm with a bandwidth of ~ 10 nm, at > 90% reflectivity and recoated in polyimide. The optical fibre containing CFBG is fixed between two micro-blocks placed circa 200 mm apart with the optical fibre magnetically clamped on them. A custom made cylindrical Teflon mould with 14 mm in diameter, 2 mm depth, with 1 mm depth grooves cut into the sides for placing the optical fibre in was used for specimen manufacture. The 10 mm CFBG is placed at the middle region of 14 mm diameter mould. After the optical fibre containing the CFBG and composite are placed in, the glass slide is placed flat on top and ready for curing. Once this process is completed the CFBG spectrum is recorded for 5 min (~ 100 data points) and averaged to produce the reference spectrum. Reference spectrum is recorded for 5 min to ensure that CFBG is properly settled within the composite and its spectrum is stable. The test composite material was then photo excited for 20 s by the curing lamp (X-Lite-II) and using the embedded CFBG at a depth circa 1 mm from the surface of the material, the dynamic PS strain is measured for another 10 min. The spectrum analyser used for measurement was Thorlabs OSA202C, which has a spectral resolution of ~ 50 pm at 1550 nm. Figure 2a,b shows the experimental arrangement.

The reflectivity-spectrum was measured by setting the maximum measured intensity from the reference spectrum to the known reflectivity of the CFBG. Both the reference and live data are linearly interpolated by a factor of 5 to artificially obtain a higher wavelength resolution for finding the edges of the CFBG region accurately, obtained by thresholding 3 dB from the maximum. However, for spectrum analysers with high spectral resolution the linear interpolation may be skipped. The extracted regions of the live data are then linearly interpolated again to fit the same wavelength domain and data size as the extracted reference. These values from the live and reference are then used to perform the IOD.

To compare the results from the CFBG sensor, an FBG array with three sensors is also embedded in the dental composite and PS strain at three locations are obtained—average strain across a 3 mm region centered at 3 mm, 7.5 mm and 13 mm of the 14 mm diameter dental composite specimen. All the experiments were conducted separately, however, the experimental conditions were kept the same to obtain comparable results. The DC of the material at the close proximity of the FBG sensor locations was also measured using FTIR technique to understand the correlation between curing lamp intensity and polymerisation strain/shrinkage.

## Results and discussion

### One dimensional strain profile of a test biomaterial (dental composite)

**Figure 3:**
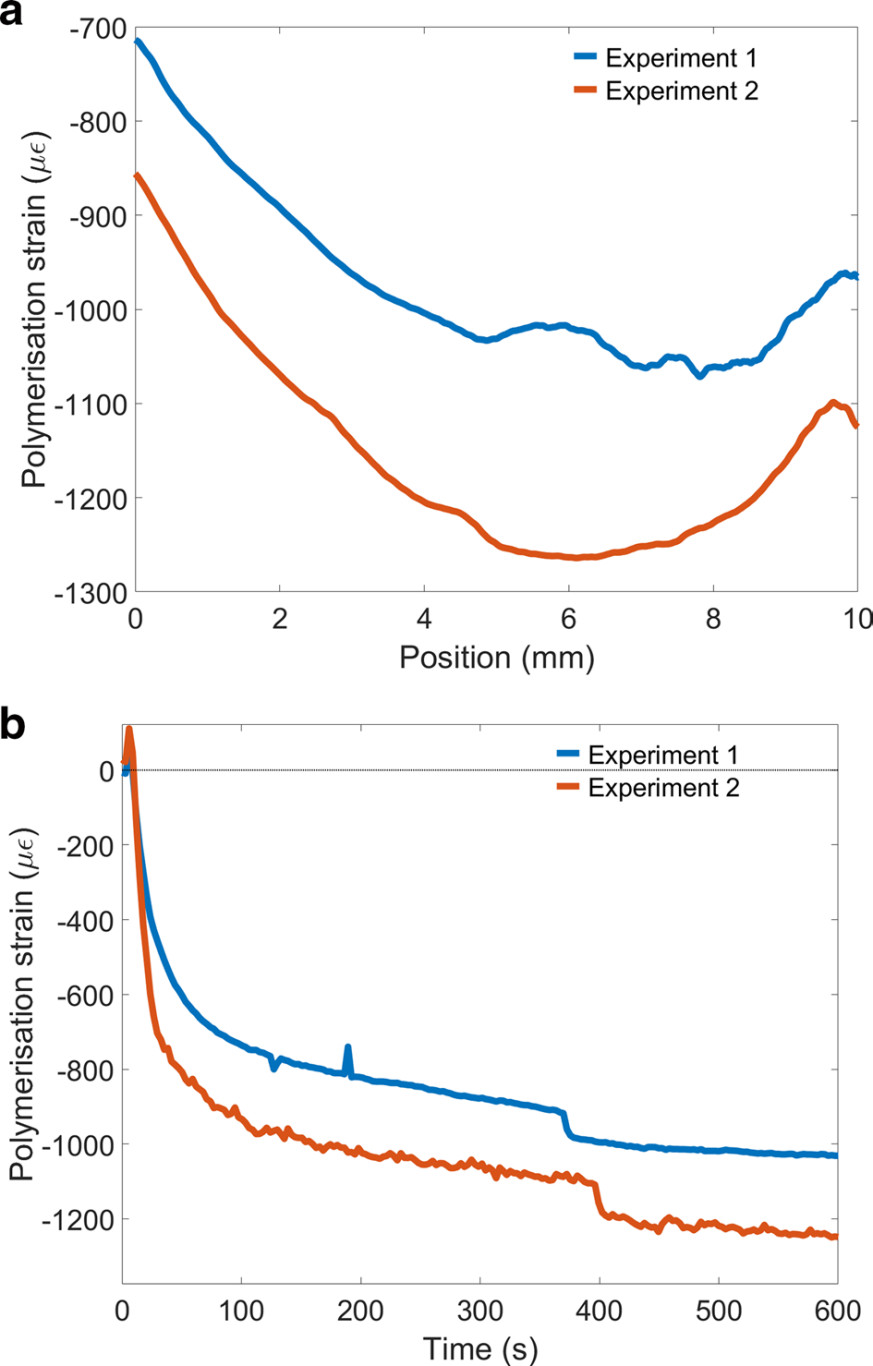
(**a**) PS strain profile of the dental composite measured using CFBG sensor, (**b**) PS strain response at 5 mm location of the dental composite specimen measured by the CFBG.

The measured polymerisation strain profile across the diagonal length of the composite from two repeated experiments conducted is shown in Fig. 3a. The measured PS strain profile shows higher shrinkage at the middle region and lower towards the edges, though an asymmetry is observed in the profile. Figure 3b shows the curing response in the middle region of the composite specimen (at 5 mm position on the CFBG). The initial spike in the result is due to a thermal rise from the curing exotherm and the curing lamp energy. Direct FBG peak wavelength shift compensation alone cannot be used to discern between temperature and strain effects and thus a temperature compensation technique would be needed to eliminate this spike, which is not considered in this study. The PS slope observed is typical of photo-cured composites, where circa 75% of the PS occurs within the first 10 min of curing^35^. Approximately a maximum of 200 με difference is observed (at 5 mm point of the CFBG) in the PS strain between the two experiments which could be due to the difference in the embedment depth of the CFBG within the composite. Multiple experiments also showed that PS strain of the same material with the same experimental setup yield similar results with an experimental error of circa 15%. However, this can be overcome by using appropriate experimental setup where the embedment depth of the CFBG can be accurately set for all experiments. Spikes and step changes in strain are also seen in the curing response (Fig. 3b), which is attributed to minor delamination/debonding/jittering of the embedded optical fibre during the curing process. However, this will only have minimal influence on the overall result and is ignored in the current study.

**Figure 4:**
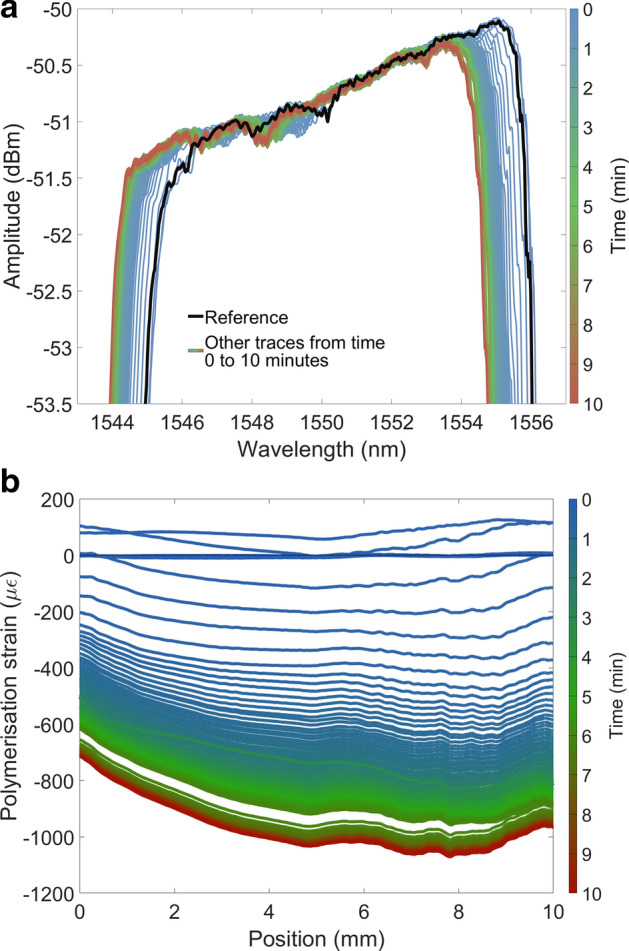
(**a**) Changes in the CFBG spectra due to PS strain, (**b**) evolution of PS strain within the dental composite for a duration of 10 min from cure initiation measured by the CFBG sensor.

The spectral changes of the CFBG during the curing process and the PS strain profile evolution within the dental composite for first 10 min is shown in Fig. 4a,b respectively. This gives an overview on how the curing process is undertaking in the entire diagonal region of dental composite, rather than the average shrinkage for the material obtained by other traditional methods. The curing rate is higher for initial phase (~ first 200 s) for the entire region and then slows down and become stable within 10 min. From this data (Fig. 4b) the curing characteristics of the material at any point along the length of the CFBG can be obtained as shown at 5 mm position in Fig. 3b.

The asymmetry in the shape of the measured PS strain profile could be attributed to a variety of causes. The CFBG region spans 10 mm whilst the marked region on the optical fibre containing the CFBGs (commercial CFBG) used were 14 mm. Though we assume the CFBG is in the centre of the marked region, the exact location of the CFBG within the fibre was unknown and its placement within the mould might be slightly off-centre with the worst case error of ± 2 mm. Another plausible cause for the asymmetry is a poor endpoint selection where the wavelengths used for the IOD process is outside the CFBG region. The tailing artefact observed at the end supports this hypothesis as well since the tailing may be from performing the IOD outside the CFBG region where there is no experimentally induced wavelength-shifts and thus simply a noise-level amount of measured strain. A poor endpoint selection also leads to a poor edge-point wavelength shift measured and thus poor endpoint values used to re-fit the curve. This issue can be solved by having short 1 mm FBG sensors at both sides of the CFBG, which be used as CFBG edge references.

**Figure 5:**
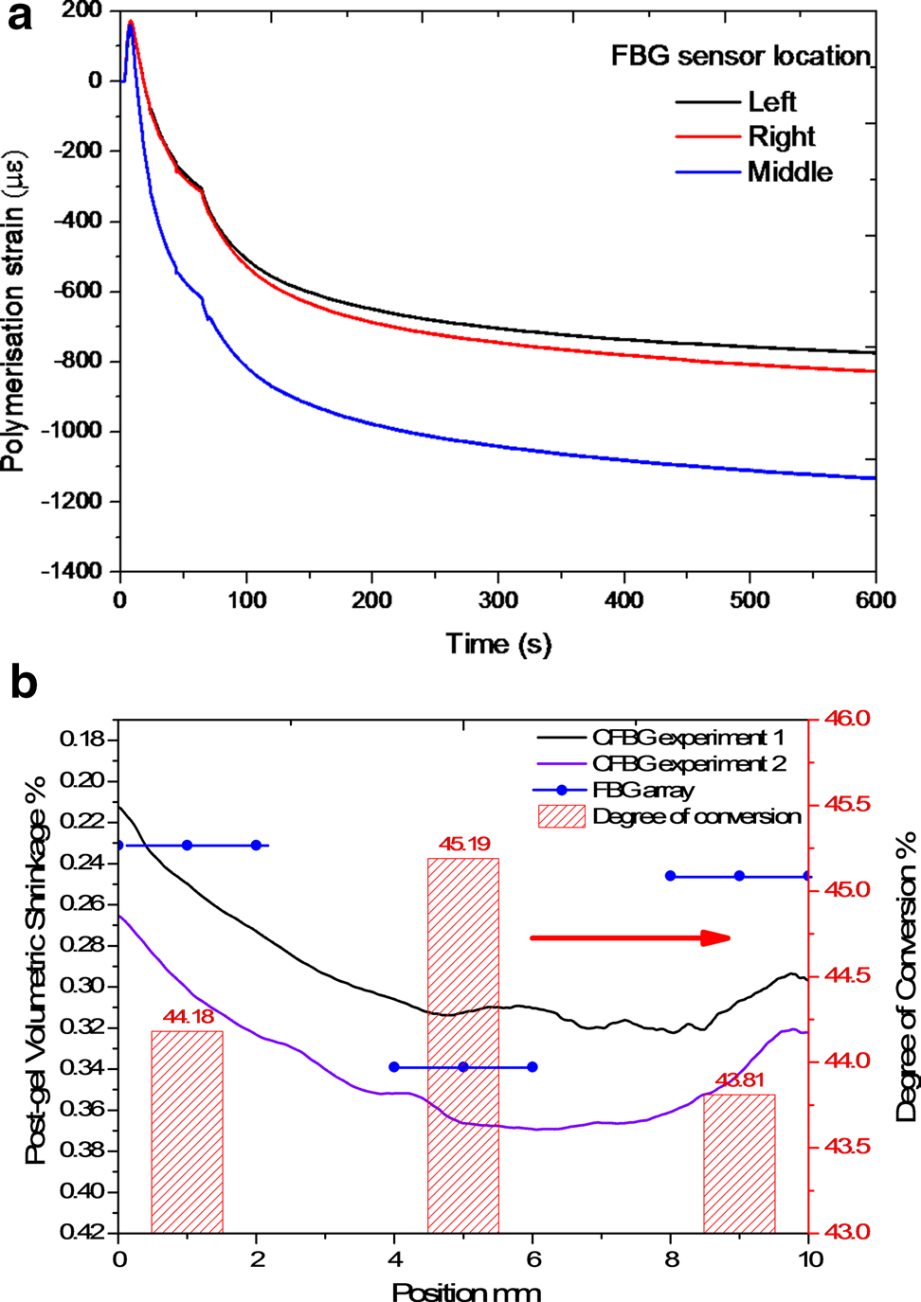
(**a**) PS strain response at three locations of the dental composite measured by the FBG array, (**b**) comparison of post-gel volumetric PS of the dental composite measured by CFBG sensor and FBG sensor array.

The PS strain results from the CFBG is also compared with that of an FBG array embedded in the dental composite where the average PS strain across a 3 mm region centered at 3 mm (left region), 7.5 mm (middle region) and 13 mm (right region) of the dental composite specimen is measured. The PS strain response measured by the FBG array is shown in Fig. 5a where the average strain centered at 3 mm and 13 mm approximately corresponds to the strain at the left (0–2 mm position) and right edges (8–10 mm position) of the CFBG respectively and strain measured by the FBG at 7.5 mm corresponds to the strain measured by centre region of the CFBG (4–6 mm position). Post-gel volumetric shrinkage of the specimen is estimated from the PS strain measured by both CFBG and FBG array sensors assuming the material is isotropic is shown in Fig. 5b. FBGs measure average post-gel volumetric polymerisation shrinkage of 0.23%, 0.34% and 0.25% for the left, centre and right regions, whereas the CFBGs recorded an average (of two experiments across the same length of the FBG) of 25%, 34% and 31% for the same region. The degree of conversion of the material at the FBG array locations was also measured and is also shown in Fig. 5b, where the PS strain is proportional to DC, which is influenced by the curing lamps’ intensity characteristics. The results from the CFBG and FBG arrays are comparable and exhibits similar trend. More accurate and precisely controlled experimental conditions such as depth of optical fibre within the material, position of the sensor would yield a more comparable result.

The results from the FBG array and CFBG demonstrate that by using a single CFBG sensor, distributed shrinkage strain profile (one dimensional/cross sectional) of the material can be obtained and can reduce the complexity associated with FBG arrays which provides similar results with less spatial resolution. Furthermore in this work only one CFBG is used as a proof-of-concept demonstration, however having multiple CFBGs, 2D or 3D strain profiles of the material can be obtained which would not be possible by any other means.

### Potential clinical benefits of the new PS profiling method

The developed approach has many potential clinical benefits. To facilitate the development of innovative dental composites/biomaterials, a better understanding of their characteristics and curing response is required, from micro to macro scales. The ability to photo-cure composite resins is based on position and orientation of the curing light and its radiant energy^2^. Currently available curing lights vary in many aspects such as different tip diameter, intensity and irradiance which will affect the resultant cure outcomes. As such the physical characteristics of the material, which are a function of the curing light characteristics, therefore, would be different in laboratory test conditions and in clinical applications. One common approach adopted by practitioners and curing light manufacturers is to increase the light intensity and irradiance, so that under any circumstances, the composite resin is photo activated well. The ISO standard limit the irradiance to 200 mW/cm^2^ for the wavelength region of 190–380 nm, however, most of the commercial curing light’s spectral distribution is beyond 380 nm and have irradiance up to 2000 mW/cm^2^ ^36,37^. This itself possess a significant health hazard to the patients and the practitioners. The excess heat generated by the curing light, and the exothermic reaction of the composite resin, will affect the tooth pulp and soft tissues^38,39^.

So simply increasing the irradiance is not the solution to the problem. The solution is to understand the response of the composite resin towards the curing light characteristics and optimise the composite-light system. Having the shrinkage profile information of the composite across specimen and its correlation with curing lamp intensity will allow the clinicians to work more informed and to modify/improve their restoration techniques which will improve their longevity.

## Conclusion

We have demonstrated an IOD based approach for interrogation of a CFBG for distributed strain measurements or strain profile mapping. As a proof of concept of the developed approach the technique was applied to PS strain profile measurement of photo-cured dental composites. The successful results show potential use of this technology for shrinkage profile mapping of polymer-based biomaterials and composites. The technique requires refinement to mitigate strain-gradient errors and further work for 2D/3D strain profiling.

## Data Availability

The data that support the findings of this study are available from the corresponding author upon reasonable request.
